# Enhanced Phytoremediation of Galaxolide Using *Lemna minor*: Mechanisms, Efficiency, and Environmental Implications

**DOI:** 10.3390/ijms26146636

**Published:** 2025-07-10

**Authors:** Aneta Sokół, Joanna Karpińska

**Affiliations:** Department of Analytical and Inorganic Chemistry, Faculty of Chemistry, University of Bialystok, Ciolkowskiego 1K Str., 15-245 Bialystok, Poland; joasia@uwb.edu.pl

**Keywords:** galaxolide stability, phytoremediation, removing efficiency, wastewater, removal products

## Abstract

This study aims to evaluate the potential of *Lemna minor* (common duckweed) for the removal of galaxolide (HHCB) from polluted water, a compound commonly used in consumer products such as perfumes and detergents. The focus was to identify the optimal conditions for removal, determine the removal efficiency, and elucidate the mechanisms involved. The experiment was conducted by cultivating *Lemna minor* using as a cultivation medium synthetic sewage and laboratory solutions (MilliQ water) containing galaxolide at two levels of concentration (1034 µg·L^−1^ and 2326 µg·L^−1^). The plants were exposed to light for 16 h a day and grown at pH 5. Removal efficiency was assessed through liquid chromatography (HPLC) with fluorescence detection (FLD). Kinetics of observed process was modelled using a pseudo-first-order equation. The study of the HHCB decay mechanism included determining the contributions to the final effect of the following processes occurring simultaneously: sorption on the plant surface, photodegradation, and uptake by Lemna. The removal efficiency (RE%) of galaxolide by *Lemna minor* was 99.7% when aqueous standard solution was used as the cultivation medium after 14 days, and between 97.8% and 98.6% in the case of wastewater samples. Sorption onto plants surface, photodegradation, and uptake by the plants were identified as the primary mechanisms for HHCB removal. Toxicity studies revealed that galaxolide exposure adversely affected *Lemna minor* growth, altering photosynthetic pigments (chlorophyll and carotenoid) levels.

## 1. Introduction

Duckweed is a plant capable of removing organic impurities and nutrients, using them to build biomass. That is why it has been applied for removing toxic nutrients and heavy metals from aquatic environments, due to its easy maintenance and resilience to severe environmental conditions [1,2]. It is characterised by a high tolerance to organic and inorganic pollutants, the ability to accumulate several contaminants simultaneously, and resistance to stressful environmental factors. In addition, duckweeds are also being studied as a source of biomass for biogas production [3,4,5,6]. Duckweeds are recommended for ecotoxicological studies as model plants because of their sensitivity, simple anatomy, and fast growth [7]. The ability of *Lemna minor* to grow in a polluted place with a considerable variability of pH (3.5 to 10.5) and temperature (7 to 35 degrees Celsius) makes them effective in the process of water cleaning—phytoremediation [8,9,10,11]. Little attention has been paid to the application of duckweed to remove musks, which are seen as a new group of bioaccumulative and persistent xenobiotics [12]. Synthetic musks are a class of semivolatile and lipophilic substances used as fragrance additives to achieve a desired scent or mask the unpleasant smell of other ingredients used in the formulation of cosmetics and household commodities [13]. They are also used in food additives, tobacco products, fish bait, herbicide formulations, and explosives [14]. Due to their low price and long-lasting flavour, synthetic musks have been widely used as fragrance ingredients in daily necessities, such as detergent, perfume, and cosmetics [15,16]. Galaxolide, a main compound from this group(1,3,4,6,7,8-heksahydro-4,6,6,7,8,8-heksametylocyklopenta[g]2-benzopyran, e.g., HHCB) [17], is a polycyclic musk with a musky-floral scent, widely produced and used in considerable quantities in industry around the world [18,19]. Due to the extensive use of HHCB in formulations of personal care products (PCPs) with high consumption, a large percentage of this compound is launched on the network of wastewater sanitation, which is introduced into the environment. Around 77% of its amount is expected to enter the sanitation system after use [20]. As galaxolide (HHCB) is frequently detected in the environment, it is suspected of having potential harmful effects on the ecosystem [21]. The presence of HHCB in surface water at levels from 0.01 to 1 μg·L^-1^ was confirmed, while its concentrations in rivers near large European cities (including the Rhine and Vltava) ranged from 100 ng·L^-1^ to 1.3 µg·L^-1^ [22]. Its concentration in raw wastewater is much higher, ranging from 1 to 50 μg·L^−1^. Some studies have recorded values as high as up to 90 μg·L^−1^ in highly urbanised areas [23]. In the case of conventional wastewater treatment (e.g., activated sludge), the efficiency of HHCB removal ranges from 60% to 90%. As a result, HHCB concentrations in treated wastewater can range from 0.1 to 10 µg·L^-1^ [20,24,25,26]. Due to its lipophilic properties, HHCB tends to bioaccumulate in aquatic organisms [27]. Studies indicate that it exhibits an estrogenic effect on the organisms of aquatic animals [28]. It is therefore important to continually research the risks associated with fragrances and report the results to regulatory authorities to better control manufacturers and warn consumers [16]. Galaxolide enters aquatic systems mainly with wastewater via wastewater treatment plants. These emerging contaminants can be removed by introducing biological treatment systems with constructed wetlands units with cultivated plants. The present study aims to investigate the effectiveness of HHCB removal by duckweed (*Lemna minor*) from polluted waters. The kinetics of HHCB disappearance were investigated, and the primary mechanism responsible for this process was determined. The toxicity of HHCB to the *Lemna minor* was included to provide vital information for the risk assessment of HHCB in the aquatic environment. The research was performed using the following two concentration levels: 0, 2.0 µmol L^−1^ (517 µg L^−1^), and 9.0 µmol L^−1^ (2326 µg L^−1^). The use of higher concentrations during HHCB removal experiments with *Lemna minor* was driven by the need to optimise purification processes and ensure effective contaminant removal even at elevated concentrations. These concentration levels also allowed for the testing of the limits of removal efficiency (RE%) of HHCB and for increasing the safety margins, thereby enhancing the development of a new method for treating HHCB using *Lemna minor*.

While duckweed has been extensively investigated for its potential in wastewater treatment, particularly for removing nutrients, heavy metals, and selected organic pollutants, there remains a notable gap regarding its use in eliminating synthetic musks such as galaxolide (HHCB). Previous studies have primarily focused on conventional contaminants. To date, no comprehensive research has examined the removal mechanisms, kinetics, and transformation products of HHCB by *Lemna minor*. Addressing this gap, our study is the first to explore in depth the capacity of *Lemna minor* to remove and metabolise galaxolide under controlled conditions and in synthetic wastewater environments.

## 2. Results and Discussion

### 2.1. Phytoremediation Process Efficacy

**Figure 1:**
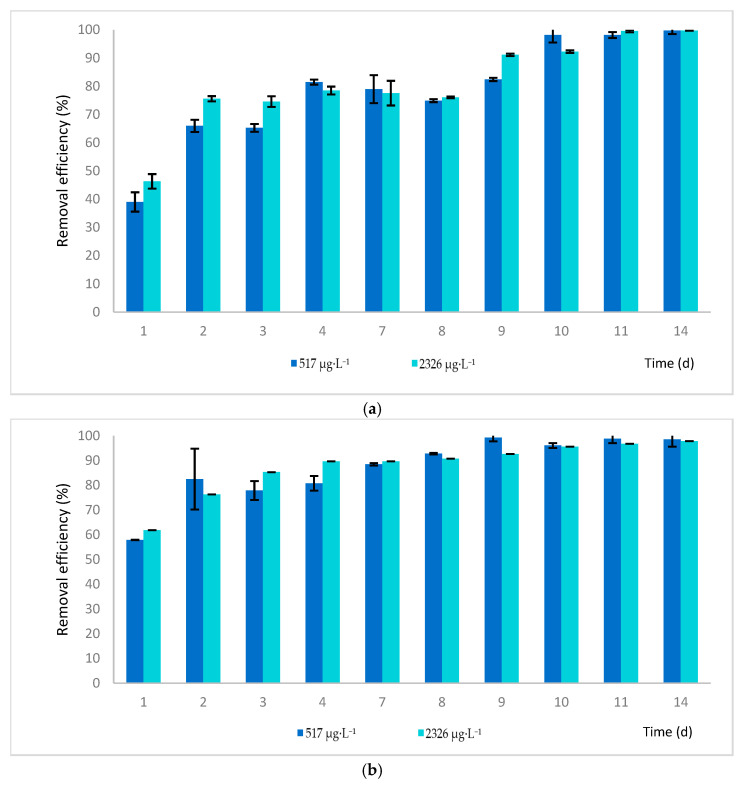
The removal efficiency of galaxolide from (**a**) the laboratory nutrient solution and (**b**) the synthetic wastewater by *Lemna minor*.

This study explores the effectiveness and underlying processes by which *Lemna minor* (duckweed) removes HHCB (galaxolide) from water. The conducted studies found that duckweed can very effectively remove galaxolide at concentrations of 517 and 2326 μg·L^−1^ after 14 days of the process (Figure 1) with an efficiency of 99.6% for laboratory nutrient solutions and in the range of 96.8% to 98.9% for synthetic wastewater. It was observed that the initial concentration of HHCB exhibits a tiny influence on the purification efficiency of the plant. The confidence intervals shown in the figures were calculated based on three independent measurements (n = 3).

The processes occurring during the removal of HHCB in the presence of plants with high accuracy can be described by the pseudo-first-order kinetics model [29]. The rate constants (k), half-lives(t_1/2_), and correlation coefficients (R^2^) which characterised removal of the HHCB by the studied processes from the laboratory nutrient solutions and the synthetic wastewater are shown in Table 1.

The obtained results confirm that the phytoremediation of HHCB by *Lemna minor* in laboratory solutions is practically independent of its initial concentration. At a 517 µg·L^−1^ concentration, the rate constant for HHCB degradation was 0.359 ± 0.012 d^−1^, corresponding to a half-life of 1.93 ± 0.61 days. The high R^2^ value of 0.935 indicates a good fit of the degradation data to the kinetic model. For the higher concentration of 2326 µg·L^−1^, the rate constant slightly decreased to 0.345 ± 0.08 d^−1^, with a corresponding half-life of 2.21 ± 0.332 days. The R^2^ value improved to 0.950, suggesting a better model matching at this concentration. At 517 µg·L^−1^, the degradation rate constant in synthetic wastewater was 0.37 ± 0.011 d^−1^, yielding a half-life of 1.87 ± 0.006 days. The R^2^ value of 0.981 indicates an excellent fit, suggesting that the degradation process closely follows the expected kinetics in this medium. However, at the higher concentration of 2326 µg·L^−1^, the rate constant dropped significantly to 0.312 ± 0.368 d^−1^, with a much longer half-life of 12.22 ± 0.028 days.

The results demonstrate that in synthetic wastewater, HHCB degrades more rapidly at lower concentrations, as indicated by the higher rate constants and shorter half-lives. The higher concentration of HHCB (2326 µg·L^−1^) resulted in a slower degradation process in synthetic wastewater, where the half-life was significantly longer. The efficiency of the removal of HHCB is influenced by both the concentration of the compound and the composition of the synthetic wastewater, which shows a more pronounced decrease in the degradation rate at higher concentrations. The lower efficiency of HHCB removal from synthetic wastewater compared to the laboratory nutrient solution may be attributed to the complexity of the wastewater matrix. Synthetic wastewater contains more organic matter and nutrients, increasing carbon availability. This richer matrix can interfere with the degradation processes, potentially inhibiting the breakdown of HHCB. The presented results are consistent with the literature, e.g., Imron et al. [30] studied the efficiency of methylene blue (MB) removal using *Lemna minor.* It was found that after 2 days of contact of the plant with the solution, the concentration of MB decreased by about 82%. Al-Nabhan [31] demonstrated that *L. minor* effectively removed Pb (88.8%), Cr, and Cd (22.22%) from water, highlighting its potential as a phytoremediation agent. Rakhshaee et al. [32] found that pre-treated *Lemna minor* effectively removed Hg(II), Cr(III), Cr(VI), and Cu(II) from aqueous solutions. Similarly, *L. minor* has been found capable of remediating petroleum hydrocarbons, as shown in the study by Kösesakal et al. [33] where the plant successfully removed aromatic hydrocarbons, suggesting its effectiveness in addressing other organic pollutants. In our previous studies [34] we showed that *Lemna* is an effective agent for removing benzotriazole UV filters from contaminated water. In the case of the tested UV filters UV-P, UV-326, UV-329, UV-328, and UV-327, efficiencies of 97, 100, 98, 99, and 99% were obtained after 14 days of cultivation, respectively. Additionally, the study by Dosnon-Olette et al. [35] revealed that *L. minor* was capable of removing pesticides such as isoproturon and glyphosate, further suggesting its applicability in remediating other organic pollutants, including HHCB. These findings collectively underscore the versatility of *L. minor* in the removal of a wide range of environmental contaminants through phytoremediation.

### 2.2. Mechanisms of Removal HHCB by Lemna minor

Based on the experiments conducted, it was observed that the removal of galaxolide from aqueous solutions can be attributed to the processes of hydrolysis, photodegradation, sorption, and plant uptake [36]. Each process contributes differently to the overall removal efficiency, highlighting the complex interactions that govern the fate of galaxolide in the environment. These processes collectively reduce galaxolide levels in aqueous environments, ensuring removal and potentially mitigating the environmental impact.

In order to determine the contribution of the individual processes to the efficiency of HHCB removal, reaction rate constants and half-life times were determined for each of them. The calculated average values of rate constants and half-lives for individual processes involved in the removal of HHCB are presented in Table 2.

The mechanism of galaxolide removal by duckweed from water and synthetic wastewater is complex and involves several processes, each contributing to the overall efficiency of the removal process. Based on the relationships described above, the percentage of contribution of various processes to galaxolide removal (HHCB) was calculated. The results show that the hydrolysis process exhibits a small impact on the efficiency of galaxolide disappearance (from 6.9% to 16.8%). Sorption has the most significant contribution to removal (from 42.0% to 47.9%), indicating that dead plant material has a high ability to retain this compound. Photodegradation (ranging from 23.6% to 29.1%) and duckweed uptake (ranging from 10.3% to 21.2%) occur at similar rates and are also important in the overall removal efficiency of HHCB. Both processes should be considered when developing wastewater treatment strategies. The uptake of galaxolide by duckweed is less effective at higher concentrations of HHCB. This is due to the deteriorating fettle of duckweed under such conditions, suggesting that maintaining the appropriate HHCB concentration in wastewater is crucial for its effective removal. In practice, wastewater treatment systems should be designed considering all four mechanisms (sorption, plant uptake, hydrolysis, and photodegradation) of HHCB removal. Particular attention should be paid to maintaining conditions that support the healthy condition of duckweed, which can contribute to increasing the overall efficiency of the process.

In summary, effectively removing galaxolide from synthetic wastewater requires understanding and optimising various mechanisms, focusing on managing conditions conducive to photodegradation and uptake by duckweed.

This information is crucial for designing phytoremediation systems, where the appropriate selection of methods, such as sorbents and plants, can significantly enhance the effectiveness of removing galaxolide from contaminated environments. These conclusions provide a better understanding of the mechanisms of galaxolide removal and can contribute to developing more effective environmental remediation strategies. The studies indicate that duckweed (*Lemna minor*) can accumulate and metabolise galaxolide (HHCB), making it a potential tool for the phytoremediation of waters contaminated with this compound. This conclusion is consistent with our previous studies, in which we showed that the removal of benzotriazole UV filters is mainly due to sorption and uptake processes by the plant [34]. The findings of Imron et al. confirm that *Lemna minor* effectively removes methylene blue dye from aqueous solutions through phytosorption (hydrogen bonding and electrostatic interaction) and biodegradation (desulfurization and denitrification) [30]. Similarly, Gatidou et al. demonstrate that benzotriazole compounds are removed through a combination of plant uptake, photodegradation, and hydrolysis, with plant uptake being the dominant process [36]. These studies emphasise that *Lemna minor* is highly effective in reducing organic pollutants from water.

These findings carry broader environmental implications as they highlight the potential of *Lemna minor* not only to reduce pollutant levels in water bodies but also to metabolise persistent synthetic compounds such as HHCB. The plant’s demonstrated tolerance to HHCB, alongside identifying a stable metabolite (HHCB-lactone), suggests a natural, sustainable pathway for environmental detoxification. Furthermore, by providing insight into the physiological stress responses of the plant, this study contributes to understanding the ecological risks of galaxolide in aquatic environments. Thus, this work aligns with the growing demand for nature-based, low-energy water treatment solutions that minimise environmental harm.

### 2.3. Identification of the Primary Metabolite Formed During the Removal of Galaxolide

During the removal of galaxolide by plants, several metabolites are formed as the compound undergoes biotransformation [37]. After 14 days of treatment with *Lemna minor* in wastewater solutions, one of the main metabolites identified was galaxolide lactone. The presence of this transformation product aligns with findings from Bester (2004), who identified HHCB-lactone as the primary oxidised metabolite of galaxolide in a German wastewater treatment plant [23].

The detection of HHCB-lactone in the solution using GC-MS (molecular weight: 272, retention index: 2206, most intense peak at *m*/*z* 257) is in the Supplementary Material (Section S1), and further confirmation by LC-MS/MS (retention time: 1.79 min, parent ion: *m*/*z* 273.8, fragment ions: *m*/*z* 256.15, 226.10, and 198.2) in the Supplementary Material (Section S1 and Figure S1) demonstrates that *Lemna minor* actively participates in the transformation of galaxolide into HHCB-lactone. This finding is consistent with the results of Su et al. (2023), who identified Galaxolidone (HHCB-lac) as the dominant transformation product in water systems, highlighting its persistence and ecological significance [38]. The biotransformation of galaxolide by *Lemna minor* suggests that aquatic plants can play a role in the removal of synthetic musk compounds from wastewater and natural waters. This aligns with research by Ding et al. (2020), which identified hydroxylation, methoxylation, methylation, ketonization, and demethylation as key metabolic pathways in HHCB degradation by microalgae [37].

These findings emphasise the role of aquatic plants in bioremediation and provide further evidence that HHCB-lactone is a stable intermediate product, frequently detected in rivers and wastewater. The ability of *Lemna minor* to metabolise HHCB into HHCB-lactone and potentially other transformation products suggests an important natural pathway for synthetic musk removal in aquatic environments.

### 2.4. Accumulation of HHCB in Lemna minor

After confirming that plants take up HHCB, the study was conducted to determine whether HHCB accumulates in plants or is metabolised. Plant material was collected on days 1, 2, 3, 4, 7, 8, 9, 10, 11, and 14 during cultivation in a medium enriched with HHCB. The plants were then subjected to extraction, and the concentration of HHCB in the obtained solutions was measured using HPLC-FLD.

This method allowed for tracking HHCB accumulation over time and detecting any changes in concentration that might indicate metabolism. Regular HPLC-FLD analysis helped determine whether HHCB was accumulating in plant tissues or being transformed into other compounds, suggesting metabolism by the plant.

**Figure 2:**
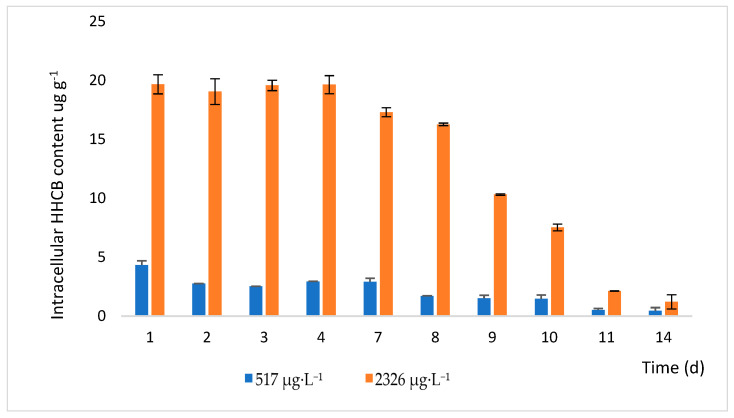
Intracellular accumulation of HHCB in *Lemna minor* tissues after exposure to contaminated wastewater. Error bars represent the standard deviation of 3 replicates.

On the first day of the experiment, an increase in intracellular HHCB was observed in *Lemna minor* when exposed to concentrations of 517 µg·L^−1^ or 2326 µg·L^−1^ of HHCB in wastewater (Figure 2).

This increase is more pronounced in the case of the higher HHCB concentration (2326 µg·L^−1^), reaching a peak content of about 19.64 ± 0.77 µg g^−1^ after 4 days. For the lower HHCB concentration (517 µg·L^−1^), HHCB content increases to about 2.93 ± 0.31 µg g^−1^ during the same period. For both HHCB concentrations, after reaching the maximal content, there is a stabilisation period between 1 and 7 days where the HHCB level remains relatively constant. After day 7, a decrease in intracellular HHCB content was observed in both cases.

For instance, the bioaccumulation of HHCB decreased from 4.33 ± 0.36 µg·g^−1^ on day 1 to 0.44 ± 0.29 µg·g^−1^ on day 14. A similar trend was observed for a solution at 2326 µg·L^−1^ HHCB exposure for *Lemna minor.* The intracellular concentration of HHCB was 19.67 ± 0.81 and 1.21 ± 0.61 µg·g^−1^, respectively, on day 1 and day 14. In both cases, there is an effective reduction in HHCB concentration in plant tissues. The decrease in HHCB content in plant tissues suggests that duckweed not only accumulates but also transforms galaxolide.

Duckweed efficiently absorbs HHCB from water, leading to a reduction in its concentration in the aquatic environment. The plant transforms the contaminant, reducing its toxicity and environmental concentration. Duckweed has significant potential in the phytoremediation of waters contaminated with galaxolide, as confirmed by studies showing the effective reduction of HHCB in plant tissues. The presented results are consistent with the literature, e.g., the research by Tatar & Öbek confirms that *Lemna minor* can accumulate high concentrations of boron, reaching up to 274 mg·g^−1^, demonstrating its strong potential for boron phytoremediation [39]. Additionally, Al-Nabhan (2022) provides evidence that *Lemna minor* accumulates and removes heavy metals such as lead (Pb), cadmium (Cd), and chromium (Cr) from polluted water, with lead showing the highest removal efficiency [31].

Furthermore, Konakci confirms that *Lemna minor* efficiently accumulates molybdenum (Mo), lead (Pb), and copper (Cu) from acidic mine water, making it a promising species for metal-contaminated water remediation [40]. These findings further validate the ability of *Lemna minor* to act as a natural biofilter in polluted environments.

The studies reviewed confirm that *Lemna minor* is a highly effective phytoremediation tool for removing various pollutants, including dyes, heavy metals, and organic contaminants, through a combination of sorption, biodegradation, and plant uptake. The efficiency of pollutant removal depends on environmental factors such as pH and ammonium concentration. These findings highlight the potential of *Lemna minor* in developing cost-effective and sustainable wastewater treatment systems.

### 2.5. Studies on the Influence of HHCB and Synthetic Wastewater on the Growth, Biochemical Composition, Stress Markers, and Antioxidant Activity of Lemna minor

**Figure 3:**
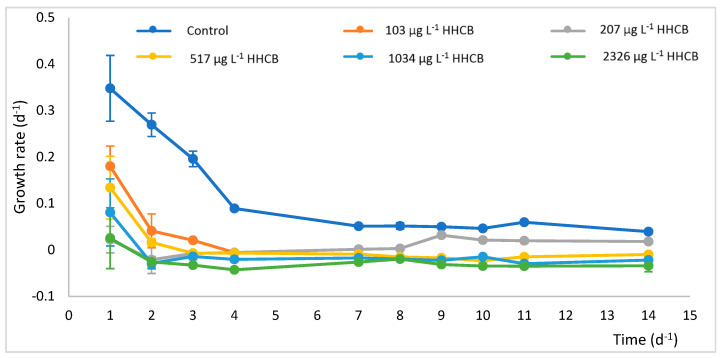
The *Lemna minor* growth rate exposed to different concentrations of HHCB. Confidence intervals were calculated based on n = 3 replicates.

**Figure 4:**
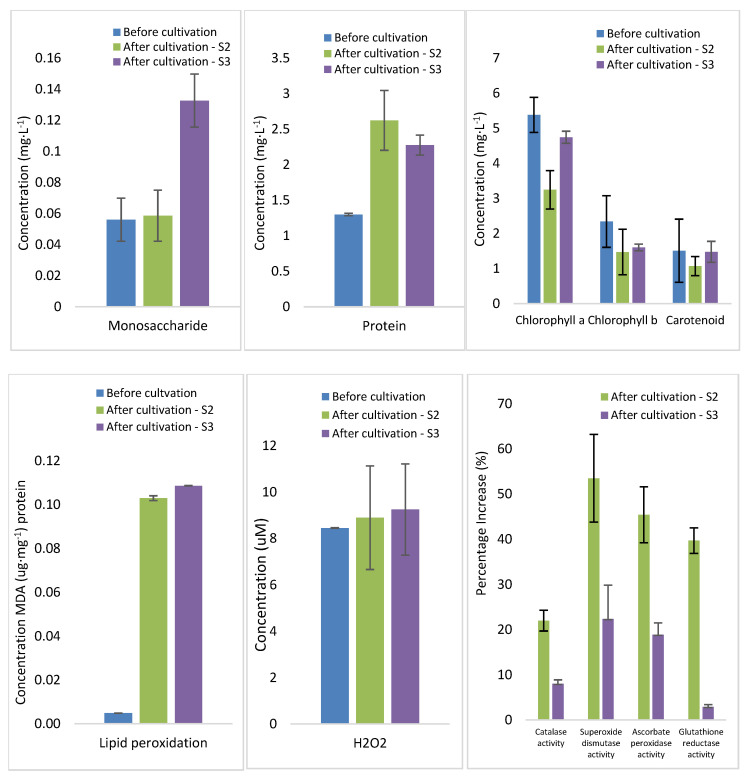
Biochemical components, stress markers, and antioxidant activity of *Lemna minor* used for wastewater purification. S2 and S3 represent *Lemna minor* samples exposed to HHCB concentrations of 517 µg·L^−1^ (S2) and 2326 µg·L^−1^ (S3), respectively, after 14 days of cultivation. Confidence intervals were calculated based on n = 3 replicates.

It is well-known that growing conditions influence the condition of plant organisms. Therefore, the impact of HHCB (ranging from 103 µg·L^−1^ to 2326 µg·L^−1^) and synthetic wastewater as a growing medium on the vital parameters of *Lemna minor* was investigated. To achieve this, the plant samples were analysed for their content of proteins, monosaccharides, photosynthetic pigments, non-enzymatic antioxidants, and enzymes involved in H_2_O_2_ metabolism before and after cultivation in the effluent wastewater, as detailed in the experimental section. The results of the plant analysis are presented in Figure 3 and Figure 4.

The control sample (not containing HHCB) shows the highest initial growth rate, which quickly drops within the first 2 days and then stabilises at a low level. All other samples containing HHCB in various concentrations (from 103 µg·L^−1^ to 2326 µg·L^−1^) show a lower growth rate than the control sample, which indicates that the presence of HHCB, regardless of the concentration, inhibits the growth of duckweed. Cultures with the highest HHCB concentrations (1034 µg·L^−1^ and 2326 µg·L^−1^) showed the lowest growth rates throughout the experiment. HHCB affects *Lemna minor* growth, with higher concentrations leading to more significant growth inhibition.

A more dramatic loss of plant biomass was observed in *Lemna minor* cultures exposed to higher concentrations of HHCB. Similar findings were reported by Basiglini et al. (2018), who showed that industrial wastewater negatively affects *Lemna minor* growth, with stronger effects observed in winter conditions [41].

After 14 days of the experiment, changes in the concentrations of sugars, proteins, assimilation pigments, and oxidative stress markers were measured in plants grown in synthetic wastewater enriched with HHCB (517 or 2326 µg·L^−1^). The highest concentration of galaxolide at which no toxic effects on duckweed organisms were observed (NOEC) was 103 µg·L^−1^. In response to stress induced by HHCB presence, there was a change in monosaccharides level. Monosaccharides, essential building blocks and critical plant energy sources, are crucial for initiating various biochemical processes. The increased monosaccharide content in *Lemna minor* exposed to wastewater and HHCB may be due to enhanced uptake of organic compounds from the environment, aligning with the findings of Walsh et al. (2020) on the influence of dairy industry wastewater composition on duckweed metabolism [42]. The total protein level was notably higher in *Lemna minor* after wastewater cultivation.

The decreasing content of chlorophylls in plant tissues can be a valuable indicator for monitoring plant nutrition and the trophic status of the environment. This reduction in chlorophyll levels can be attributed to several factors, including inhibiting chlorophyll biosynthesis, the breakdown of pigments or their precursors, and destroying chloroplast membranes due to lipid peroxidation without sufficient antioxidants. Similar effects were reported by Sun et al. (2019), who demonstrated that exposure to decabromodiphenyl ether led to oxidative stress, decreased photosynthetic efficiency, and increased antioxidant enzyme activity in *Lemna minor* [43].

This observation aligns well with findings of increased hydrogen peroxide (H_2_O_2_) rates and levels of lipid peroxidation occurring, as indicated by increased malondialdehyde (MDA) levels in *Lemna minor* biomass cultures after cultivation in wastewater.

Overproduction of ROS is a well-recognised plant response to abiotic stress factors; furthermore, MDA production under adverse environmental conditions is a reliable indicator of free radical formation in biological systems [44]. The rise in lipid peroxidation and H_2_O_2_ levels suggests the plant experienced enhanced oxidative stress. In response to ROS production, plants activate enzymatic antioxidant defence mechanisms, including low molecular mass scavengers, to eliminate these highly reactive molecules.

The adaptation of *Lemna minor* to grow in polluted aquatic environments rich in the toxic compounds present in wastewater is evidenced by the increased activities of antioxidant enzymes catalase, superoxide dismutase, ascorbate peroxidase, and glutathione reductase. The activity of antioxidant enzymes suggests effective antioxidant defence associated with removing reactive oxygen species. A comparable phenomenon was observed in the study by Huang et al. (2013), which reported increased superoxide dismutase (SOD) activity and changes in chlorophyll content in *Lemna minor* under ammonium-induced stress [45]. Similarly, Sun et al. (2019) documented elevated antioxidant enzyme activity in *Lemna minor* in response to organic pollutants [43].

In summary, the experiment’s results emphasise the comprehensive impact of HHCB on *Lemna minor* plants and their ability to adapt and respond to stress. Understanding these mechanisms is crucial for phytoremediation research and assessing the risk of galactoside contamination in water bodies. The adaptive mechanisms of this plant highlight its potential for the phytoremediation of water contaminated with toxic substances.

## 3. Materials and Methods

### 3.1. Chemicals and Reagents

Galaxolide (Sigma-Aldrich, Darmstadt, Germany), a stock solution at 2 g·L^−1^, was prepared in ethanol and stored in the dark at −4 °C for two weeks. Honeywell Chemicals supplied acetonitrile and methanol of HPLC grade. Chemicals (including KNO_3_, K_2_HPO_4_, KH_2_PO_4_, MgSO_4_·7H_2_O, CaNO_3_·4 H_2_O, H_3_BO_3_, EDTA-Na, MnCl_2_·4 H_2_O, ZnSO_4_·7 H_2_O, NaMoO_4_·2 H_2_O, and FeCl_6_ 6 H_2_O) used for the *Lemna minor* growth medium preparation were purchased from POCh, Gliwice, Poland. Chemicals (including peptone, meat extract, urea, KH_2_PO_4_, NaCl, CaCl_2_·2H_2_O, and MgSO_4_·7H_2_O) used for synthetic sewage medium preparation were purchased from Sigma-Aldrich, Darmstadt, Germany. The methods for preparing the growth medium and synthetic wastewater are presented in Sections S2 and S3, Tables S1–S3 (Supplementary Material). Other reagents were ethanol with a purity higher than 96%, concentrated acetic acid (POCh, Gliwice, Poland), dichloromethane, and hexane (Sigma-Aldrich, Darmstadt, Germany). Glassware materials were washed using only organic solvents and water.

### 3.2. Apparatus

The chromatographic measurements were performed by applying a liquid chromatograph with an FLD detector. The chromatographic separations were run on C18 column Hypersil Gold (250 mm × 4.6 mm, 5 µm) using a Thermo Scientific UltiMate 3000 HPLC instrument (Dionex, Sunnyvale, CA, USA). The HPLC system consisted of a pump, autosampler, column compartment, and fluorescence detector (Thermo Scientific Dionex, Sunnyvale, CA, USA) and was controlled by Thermo Scientific Dionex Chromeleon chromatography data system (CDS) software and ImageJ software, version 7.2.

GC/MS analysis was carried out using an HP 6890 gas chromatograph with an electronic pressure control device connected to a mass spectrometric detector MSD 5973 (electron impact source and quadrupole analyser, Agilent Technologies, Santa Clara, CA, USA) equipped with an HP-5MS column (5% phenyl, 95% methylsiloxane) with a length of 30 m and an i.d. of 0.25 mm, coated with a 0.25 μm thick film, and using a split/splitless injector.

The chromatographic measurements were performed by applying a liquid chromatography−tandem mass spectrometry system (Shimadzu LC-MS/MS-8040, Kyoto, Japan) consisting of a triple quadrupole mass spectrometer with turbo ion spray ionisation source in the positive ion mode, pump (Shimadzu LC30AD, Kyoto, Japan), thermostat column oven (Shimadzu CTO-20AC, Kyoto, Japan), autosampler (Shimadzu SIL-30AC, Kyoto, Japan), degasser (Shimadzu DGU-20A5R, Kyoto, Japan), and nitrogen generator (Shimadzu Peak Scientific NM32LA, Kyoto, Japan). 

### 3.3. Removal Effectiveness and Toxicity Experiments

The HHCB solutions at nominal concentrations of 0, 2.0 µmol L^−1^ (517 µg L^−1^), and 9 µmol L^−1^ (2326 µg L^−1^) were applied for all experiments. *Lemna minor* was obtained from a natural water reservoir in the commune of Kurowo (53°06′17″ N 22°47′26″ E), a village close to Bialystok city, in an agricultural ecological area characterised by low levels of industrial pollution, abundant green spaces, and a well-preserved natural landscape. It is located in the buffer zone of the Narew Landscape Park. This region is part of a broader conservation effort aimed at protecting biodiversity and maintaining water quality, making it an ideal location for studying the phytoremediation potential of *Lemna minor* in undisturbed environmental conditions. Sampling locations were selected based on water quality parameters, vegetation density, and minimal human disturbance to ensure representative conditions. Using pre-sterilised glass containers, *Lemna minor* was gently scooped from the water surface to avoid damage to plant tissues. A small volume of the surrounding water was included to maintain the natural growth medium and prevent physiological shock during transport. Upon arrival at the laboratory, the plants were rinsed with deionized water to remove external contaminants. They were then cultivated for two weeks in controlled conditions on a sterile growth medium [46]. Before the experiment, an appropriate mass of the plants was rinsed three times with distilled water to remove impurities. Laboratory cultures were grown in sterile glass vessels covered by transparent foil (without touching the solution) to protect the medium against evaporation. Every vessel contained 100 mL of growth medium or synthetic sewage solution and 2.0 g of studied plant. Experimental solutions were prepared by adding the appropriate amount of HHCB stock solution to the growth or synthetic-sewage medium. The tests at each concentration were performed in triplicate. All experiments have been performed under the same controlled conditions in a phytotron at 22 ± 0.5 °C with a day/night cycle 16/8 (fluorescence lamp, photon flux of 50 μmol/m^2^/s). A detailed schematic of the phytoremediation setup is provided in the Supplementary Material, Figure S2.

Samples of growth medium or synthetic sewage solution 2 mL were collected and analysed after 1, 2, 3, 4, 7, 8, 9, 10, 11, and 14 days of cultivation. The collected samples underwent extraction prior to chromatographic analysis using HPLC-FLD. Plants were also collected to evaluate vital parameters and measure the HHCB concentration in plant tissues. Control samples were prepared in the same way but without the addition of HHCB solutions. Each analysis was repeated three times.

Based on the measured HHCB concentrations, the removal efficiency (RE%) was calculated using the following formula:

(1) $$RE\%=\left(1-\frac{C}{C_{0}}\right)\cdot 100$$

where C_0_ is the measured concentration of HHCB (µg·L^−1^) at the beginning of the experiment and C is the concentration at the end of the appropriate stage of the purification process, respectively [36]. The same equation was also used to calculate the removal efficiency over time, using C_0_ as the HHCB concentration (µg·L^−1^) at t = 0 days and C_t_ as the concentration at the t = 1, 2, 3, 4, 7, 8, 9, 10, 11 and 14 days, respectively.

The growing experiments were performed for 14 days. The inhibition growth assessment was performed by calculating the dry weight of duckweed tissues. In brief, an equivalent number of plants were filtered through a 0.45 μm membrane and weighed. Subsequently, the duckweed sample was dried in an oven at 105 °C until a constant dry mass was obtained and then weighed again. The difference in mass was used to determine the dry weight of the duckweed tissues.

### 3.4. Kinetics and Mechanisms of Degradation

At the same time, additional experiments under different conditions were realised to explain the fate of HHCB during its removal by floating plants and to establish the participation of abiotic and biological processes such as hydrolysis, photodegradation, sorption onto plant external surface, and plant uptake in this phenomenon. Hydrolysis involves the chemical breakdown of galaxolide due to its reaction with water. Photodegradation refers to the decomposition of galaxolide when it is exposed to light, particularly sunlight. Sorption processes that occur in a natural aqueous environment involve the adhesion of galaxolide molecules onto the surfaces of sediment particles and onto the external surface of plant bodies, which can remove them from the aqueous phase. In the presented laboratory studies, the influence of sorption on plants on the kinetics of HHCB disappearance was examined. Plant uptake is the process by which plants absorb galaxolide from water through their roots, leading to its accumulation within plant tissues.

To identify processes responsible for the HHCB elimination, experiments were also carried out without plants under dark conditions, without plants with exposure to light, and with dead plants obtained through a two-day exposure to sodium azide at a concentration of 2 g·L^−1^ [29]. In total, four separate experiments were carried out under different conditions (1–4, Table 3). The interactions between these processes were not taken into account.

The processes occurring during the removal of HHCB in the presence of plants with high accuracy can be described by the following pseudo-first-order kinetics [29]

C_t_ = C_0_e^−kt^ (2)

(3) $$\;t_{1/2}=\frac{ln2}{k}$$

where C_t_ and C_0_ (µg·L^−1^) are the HHCB concentrations at time t and t = 0, respectively, k (d^−1^) is the removal rate constant for each experiment conducted with a *Lemna minor* and under specific conditions, and t_1/2_ is the relevant half-life (days).

The rate constant of hydrolysis (k_hydrolysis_), photodegradation (k_photodegradation_), sorption (k_sorption_), and plant uptake (k_plant uptake_) were calculated using Equations (4)–(7), with the assumption that there were no mutual interactions between these mechanisms. The equations are as follows [29]:

k_hydrolysis_ = k_1_ (4)

k_photodegradation_ = k_2_ − k_1_ (5)

k_plant uptake_ = k_3_ − k_2_ − k_sorption_ (6)

k_sorption_ = k_4_ − k_1_ (7)

where k_1_, k_2_, k_3_, and k_4_ are the average values of the rate constants for the individual processes involved in removing HHCB, respectively.

### 3.5. Extraction and Quantification of HHCB in Growth Media

At the start of experiments, and after 1, 2, 3, 4, 7, 8, 9, 10, 11, and 14 days of cultivation, appropriate volumes of laboratory medium or synthetic sewage solutions were collected to determine HHCB concentration. The collected samples were subjected to liquid–liquid extraction (LLE) (Supplementary Material, Figure S3). A total of 2 mL of taken solutions were used (Supplementary Material, Figure S4). Two calibration curves were constructed: the one obtained quantitatively introduced into the test tube and extracted three times with 2 mL hexane each time (Rotator Multi Bio RS-24, Biosan, Riga, Latvia). The organic extracts were combined and evaporated to dryness in a concentrator (Concentrator plus™, Vacufuge plus Eppendorf, Hamburg, Germany) [37]. The residue was reconstituted with 1 mL mobile phase, filtrated through a 0.22 μm membrane filter (PTFE), and subjected to chromatographic analysis (HPLC-FLD). The injection volume was 10 µL and the flow rate was 1.0 mL min^−1^. The following isocratic mobile phase composition was adopted: acetic acid 0.07% in acetonitrile and acetic acid 0.09% in water (4:1). The excitation wavelength of the fluorescence detector was set to 280 nm. The emission wavelength of the fluorescence detector was set to 310 nm. The total run time was 12 min, and the HHCB peak showed a retention time of 9.97 min (supplementary by a given extraction procedure using growth medium solutions and the second by diluting the HHCB solution in a synthetic sewage medium at concentrations ranging from 2 to 2500 µg·L^−1^). The calibration curves were constructed by plotting the peak areas against the HHCB concentration (n = 3). Under the optimal detection conditions, the validation parameters of the developed method, such as linearity, detection limit, precision, repeatability, and others, were determined (Supplementary Material, Table S4). The detection limit (LOD) and limit of quantification (LOQ) of the investigated compound were counted using the equations, and we obtained LOD = 3.3·s⁄a and LOQ = 10·s⁄a (s: standard deviation, a: slope of the calibration curve). The elaborated chromatographic method of HHCB determination is characterised by low LOQ and LOD values equal to 2 and 0.6 µg·L^−1^, respectively. The method’s precision was evaluated by analysing six replicates of samples containing 517 µg·L^−1^ of HHCB (Supplementary Material, Table S5). The reproducibility was obtained by preparing three independent calibration graphs on three different days, resulting in an average slope of 70 and 107. All the materials and reagents used in the analysis were proved to be interference-free by performing two extraction blanks, for which no HHCB was detected (below LOD).

### 3.6. Extraction and Quantification of HHCB in Plant Tissues

Plant material collected on days 1, 2, 3, 4, 7, 8, 9, 10, 11, and 14 of cultivation in the HHCB-enriched medium was filtered from the remaining liquid using cellulose fibre filters and then air-dried to remove any residual water. After that, 0.1 g plant material was introduced into 10 mL test tubes, and a solvent mixture of methanol and dichloromethane in a volume ratio of 2:1 with a total volume of 6 mL was added. The test tubes were sealed and sonicated (Bandelin SONOREX DIGITEC DT 102H) for 1 h at room temperature, 35 kHz ultrasound frequency, and 230 W power [37]. Afterwards, the solution was passed through a 0.45 µm polytetrafluoroethylene(PTFE) filter (13 mm, 0.45 μm, Chromafil Xtra, Macherey–Nagel, Düren, Germany) and evaporated to dryness in a concentrator (Concentrator plus™, Vacufuge plus Eppendorf) for 60 min. The dry residue was dissolved in 1 mL of mobile phase and analysed by HPLC-FLD.

### 3.7. Analysis of Biochemical Components, Stress Markers, and Antioxidant Activity in Lemna minor

The methods for conducting subsequent experiments that are part of toxicity studies are shown in the Supplementary Material (Sections S4–S8).

All toxicity experiments were performed in triplicates in sterile, glass vessels containing 100 mL of growth medium [46] or synthetic sewage solution and 2 g of studied plant per dish. All other conditions, temperatures, light exposure times, and photon fluxes were the same (2.2). The cultures of *Lemna minor* were treated with HHCB in the range of 0.4 (103 µg L^−1^)—9 µmol L^−1^ (2326 µg L^−1^). After 7 and 14 days of treatment, the analysis of growth and selected biochemical parameters was performed. Measuring biomass, pigments, protein, and monosaccharides is necessary for plant research because those parameters constitute a primary indicator of plant responses to stress conditions. The test at each concentration was performed in triplicate.

## 4. Conclusions

The provided experiments proved that using *Lemna minor* in the phytoremediation of water contaminated with galaxolide (HHCB) is highly effective. After 14 days of contact, the removal efficiency of HHCB from water samples was 99.7%, and from wastewater it ranged from 97.8% to 98.6%. These results highlight the particular effectiveness of *Lemna minor* in addressing emerging contaminants like HHCB.

Many processes are responsible for the effective removal of galaxolide (HHCB) from synthetic wastewater by duckweed, including sorption, photodegradation, and uptake by duckweed. Each of these processes plays a crucial role in the overall effectiveness of HHCB removal. Duckweed (*Lemna minor)* demonstrates the significant capability to accumulate and metabolise galaxolide. Studies indicate that after 14 days duckweed effectively reduces HHCB concentrations in its tissues, confirming its potential in the phytoremediation of HHCB-contaminated waters.

The effectiveness of duckweed in HHCB removal is closely dependent on environmental conditions, especially HHCB concentrations in wastewater and light availability. Maintaining optimal growth and health conditions for duckweed is crucial for maximising the removal efficiency of HHCB. In addition to demonstrating high removal efficiency, the study evaluated the physiological effects of HHCB on duckweed. Changes in chlorophyll and carotenoid levels indicated that HHCB at certain concentrations can exert stress on the plant, potentially affecting its remediation performance. Establishing the threshold concentrations that allow for efficient removal without compromising plant health is thus crucial for practical applications.

The findings underline the importance of considering diverse removal mechanisms and optimising conditions favourable for photodegradation and uptake by duckweed when designing wastewater treatment systems. Such an approach ensures comprehensive and effective HHCB removal from synthetic wastewater. Duckweed’s ability to accumulate and metabolise HHCB opens new possibilities for sustainable remediation strategies in HHCB-contaminated environments. While the results are promising, it is important to note that experiments were conducted under controlled conditions. Real-world environments may present additional challenges, such as variable temperatures, nutrient levels, and organic matter content, which could influence removal efficiency. Despite these limitations, *Lemna minor* exhibited strong resilience and consistent performance, suggesting its potential for real-world application in wastewater treatment systems.

Integrating duckweed-based phytoremediation with traditional wastewater treatment technologies can lead to more efficient and environmentally friendly water remediation strategies. The use of *Lemna minor* as an efficient and eco-friendly method for purifying water and wastewater from galaxolide fits nicely into the trend of saving water and energy as it is efficient, simple, and cost-effective. Its ability to remove HHCB even in the presence of complex synthetic wastewater matrices positions it as a viable and sustainable alternative or a complementary solution to conventional wastewater treatment methods. This approach aligns with global trends promoting low-energy, nature-based treatment solutions and underscores the strategic role of duckweed in advancing green and resource-efficient water management practices.

Despite the promising results, several limitations of this study should be considered. Firstly, the experiments were conducted under controlled laboratory conditions that may not fully reflect real environmental variability, such as fluctuating temperatures, natural sunlight, or interactions with other organisms and pollutants. Secondly, toxicity assessments showed that HHCB may impair duckweed growth at higher concentrations, potentially reducing its remediation efficiency. These findings underscore the importance of optimising operational parameters to maintain plant health. Future research should explore long-term performance in real wastewater systems and examine how duckweed-based phytoremediation can be effectively integrated with conventional treatment technologies. Ultimately, this study lays the groundwork for scalable, sustainable treatment methods targeting emerging contaminants like synthetic musk.

## Figures and Tables

**Table 1:** Rate constants (k), half-lives(t_1/2_), and correlation coefficients (R^2^) determined during the removal of HHCB experiments from water and wastewater.

Test Solution	HHCB Concentration (µg·L^−1^)	k (d^−1^) ± SD; (n = 3)	t_1/2_(d) ± SD; (n = 3)	R^2^
Laboratory nutrient solution	517	0.359 ± 0.012	1.93 ± 0.61	0.935
	2326	0.345 ± 0.08	2.21 ± 0.332	0.950
Synthetic wastewater	517	0.37 ± 0.011	1.87 ± 0.006	0.981
	2326	0.312 ± 0.368	12.22 ± 0.028	0.864

**Table 2:** Calculated values of hydrolysis rate constant (k_hydrolysis_), photodegradation rate constant (k_photodegradation_), sorption rate constant (k_sorption_), plant uptake rate constant (k_uptake_), and correlation coefficients (R^2^) in experiments conducted with HHCB.

Test Solution	HHCB Concentration (µg·L^−1^)	k_hydrolysis_ (d^−1^); (n = 3) R^2^	k_photodegradation_ (d^−1^); (n = 3) R^2^	k_sorption_ (d^−1^); (n = 3) R^2^	k_uptake_ (d^−1^); (n = 3)
Laboratory nutrient solution	517	0.025 0.934	0.105 0.949	0.153 0.861	0.076
	2326	0.051 0.975	0.083 0.964	0.148 0.858	0.063
Synthetic wastewater	517	0.036 0.983	0.105 0.904	0.159 0.898	0.070
	2326	0.053 0.977	0.075 0.921	0.151 0.858	0.033

**Table 3:** Conditions for conducting experiments determining the mechanisms responsible for the removal processes.

Experiment	T (°C)	Mechanism of Degradation	Light/Presence of *Lemna minor*
1	22 ± 2	Hydrolysis	Dark/Not
2		Hydrolysis + Photodegradation	Fluorescent lamp/Not
3		Fitodegradation (Hydrolysis + Photodegradation + Sorption + Plant uptake)	Fluorescent lamp/*Lemna minor*
4		Hydrolysis + Sorption	Dark/dead *Lemna minor* (1 g·L^−1^ NaN_3_, five days)

## Data Availability

Data is contained within the article and supplementary material.

## Supplementary Materials

The following supporting information can be downloaded at: https://www.mdpi.com/article/10.3390/ijms26146636/s1. References [47,48,49,50,51,52,53,54,55,56] are cited in the supplementary materials.
