# Supplementary material for: Enhanced Phytoremediation of Galaxolide Using Lemna minor: Mechanisms, Efficiency, and Environmental Implications
Source: Int J Mol Sci. 2025 Jul 10;26(14):6636. doi: 10.3390/ijms26146636 (PMC12294896; doi:10.3390/ijms26146636)
Supplement: Supplementary file 1 [file ijms-26-06636-s001.zip › ijms-3686972-supplementary.pdf]

Supplementary material for

Enhanced Phytoremediation of Galaxolide Using Lemna Minor: Mechanisms, Efficiency, and Environmental Implications

Aneta Sokół*, Joanna Karpińska

Department of Analytical and Inorganic Chemistry, Faculty of Chemistry, University of Bialystok, Ciolkowskiego 1K Str., 15-245 Bialystok, Poland; joasia@uwb.edu.pl

* Correspondence: a.sokol@uwb.edu.pl

S1. Chromatographic analysis of the primary metabolite formed during the removal of galaxolide, with GC-MS and LC-MS/MS technique

Because of the adverse effects that the aqueous matrix has on GC analysis, the conducted GC-MS analysis was followed by isolation (i.e. dispersive liquid-liquid microextraction - DLLME) of the studied compounds. The applied procedure was as follows: 5.00 mL of the aqueous solution was placed in a 10 mL glass tube with a conical bottom screw cap. Next, 620 μL of methanol (as dispersing solvent) containing 250 μL of chloroform (as extraction solvent) was quickly injected. After that, the mixture was shaken gently until a cloudy solution was obtained, and then it was centrifuged for 7.5 min at 4000 rpm. After centrifugation, the organic phase was separated from the aqueous phase and evaporated to a volume of 20 μL under a nitrogen atmosphere. The described DLLME procedure was applied for isolation compounds after the phytoremediation process prior to GC-MS analysis.

- GC-MS analysis

The injector worked in the splitless mode at a temperature of 250 °C. The he-lium of 99.99% purity was used as a carrier gas at a flow rate of 1.7 mL·min^{-1}. The temperature of the oven was programmed to 150 °C (1 min hold) and was increasing at a rate of 15 °C min^{-1} to 280 °C, maintaining finally the maximum temperature for 10 min. The total run time was 32 min. The MS detector worked under the following conditions: temperature of the ion source 230 °C, the temperature of the quadrupole 150 °C, the temperature of the transfer line 280 °C, mass range (m/z)50–400.

- LC-MS/MS analysis

The LC-MS/MS analysis of HHCB lactone after extraction was performed on a Kinetex C-18 (50 mm × 2.1 mm, 1.7 μm) column using a mobile phase consisting of 0.1% formic acid and methanol (4:1 v/v) at a flow rate of 0.2 mL·min^{-1}. The injection volume was 5μL. The total run time was equal 4 min and characteristic peak of HHCB was observed at retention time of 0.405 and 0.437 min. The parameters of mass spectrometer analysis were as follows: the collision gas (argon), collision cell gas pressure 230 Pa, the flow rate of drying gas (nitrogen) 15 L min^{-1}, and nebulizing gas (nitrogen) 3 L·min^{-1}. Multiple reaction monitoring (MRM) mode was used to study parent → product ions (m/z) transitions for HHCB and lactone HHCB in ESI positive ionization: 258.10 → 228.00 (collision energy 33 V), 258.10 → 58.10 (collision energy 22 V), and 258.10 → 240.20 (collision energy 20 V) – HHCB and 273.80 → 256.15 (collision energy 20V), 273.80 → 226.10 (collision energy 35 V), and 273.80 → 198.20 (collision energy 41 V) – lactone HHCB.

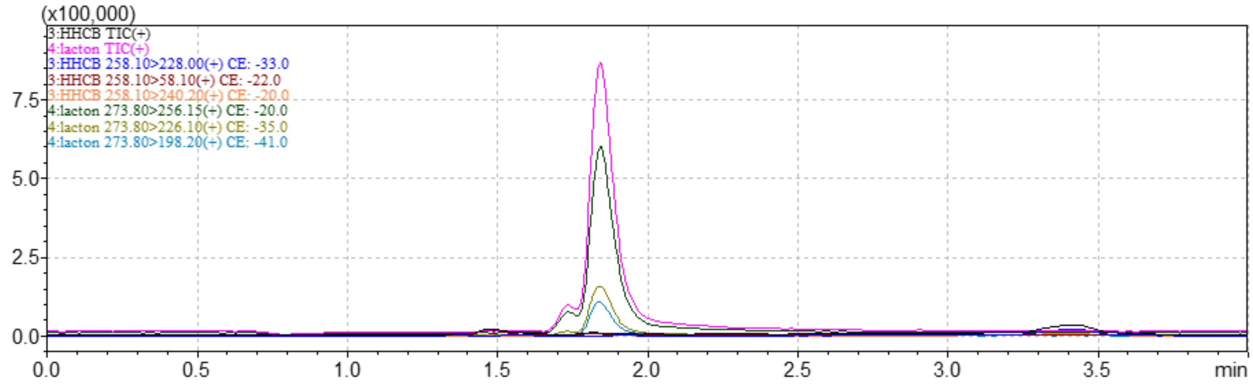

Figure S1. Chromatogram of HHCB (2326 μg·L⁻¹) after removal by *Lemna minor* from wastewater (after14 days)

S2. Preparation of growth medium (modified Steinberg medium, according to ISO 20079)

The growth medium was prepared using the substances listed in Tables 1 and 2. The medium was prepared in a 1 L flask. Initially, approximately 0.9 L of water was added to avoid precipitation. Then, 20 mL each of solutions I, II, and III were taken. After that, 1 mL each of stock solutions IV, V, VI, VII, and VIII were added and mixed. The pH of the medium was checked using a pH meter (range 5.5 ± 0.2). If the pH deviated from the recommended range, a small amount of HCl or NaOH solution was added. Finally, the volume was brought up to the mark with water in accordance with Commission Regulation (EC) No. 761/2009 of 23 July 2009.

Table S1 Stock Solutions – Macronutrients for the Preparation of Steinberg Medium

**Macronutrients**	
**Solutions**	**Concentration (g·L⁻¹)**
**Stock Solution I**	
KNO_3	17.50
KH_2PO_4	4.50
K_2HPO_4	0.63
**Stock Solution II**	
$MgSO_4 \cdot 7\ H_2O$	5.00
**Stock Solution III**	
$Ca(NO_3)_2 \cdot 4\ H_2O$	14.75

Table S2 Stock Solutions – Micronutrients for the Preparation of Steinberg Medium

**Micronutrients**	
**Solutions**	**Concentration** ($g \cdot L^{-1}$)
**Stock Solution IV**	
H_3BO_3	120.00
**Stock Solution V**	
$ZnSO_4 \cdot 7\ H_2O$	180.00
**Stock Solution VI**	
$Na_2MoO_4 \cdot 2\ H_2O$	44.00
**Stock Solution VII**	
$MnCl_2 \cdot 4\ H_2O$	180.00
**Stock Solution VIII**	
$FeCl_3 \cdot 6\ H_2O$	760.00
Disodium EDTA, dihydrate	1500.00

The medium was sterilized in an autoclave at 121°C for 20 minutes. Store the prepared substrate at 4°C until use. preparation

S3. Preparation of synthetic wastewater

Synthetic wastewater was prepared in sterilized 1 L flasks. The preparation of the solutions began with the preparation of the micronutrient solution. First, 0.5 L of deionized water was added to the flask, followed by the weighed micronutrients (Table S3). The mixture was stirred thoroughly and then topped up with water to a volume of 1 L. In the next step, approximately 500 mL of deionized water was added to a second flask, and the necessary macronutrients were weighed out. Finally, 0.6 mL of the micronutrient solution was added to the flask containing the macronutrients, and the mixture was topped up with water to the mark and stirred well [47].

Table S3 Macronutrients and Micronutrients for the preparation of synthetic wastewater

**Macronutrients**	**Concentration** ($mg \cdot L^{-1}$)	**Micronutrients**	**Concentration** ($g \cdot L^{-1}$)
CH_3COONH_4	93.75	EDTA	10.00
NH_4Cl	87.70	$FeCl_3 \cdot 6\ H_2O$	1.50
KH_2PO_4	26.70	H_3BO_3	0.15
$MgSO_4 \cdot 7\ H_2O$	9.00	$CuSO_4 \cdot 2H_2O$	0.03
$CaCl_2$	4.72	KI	0.18

KCl	36.00	$MnCl_2 \cdot 4 H_2O$	0.12
**Micronutrient Solution** 0.60 mg·L^{-1}		$Na_2MoO_4 \cdot 2 H_2O$	0.06

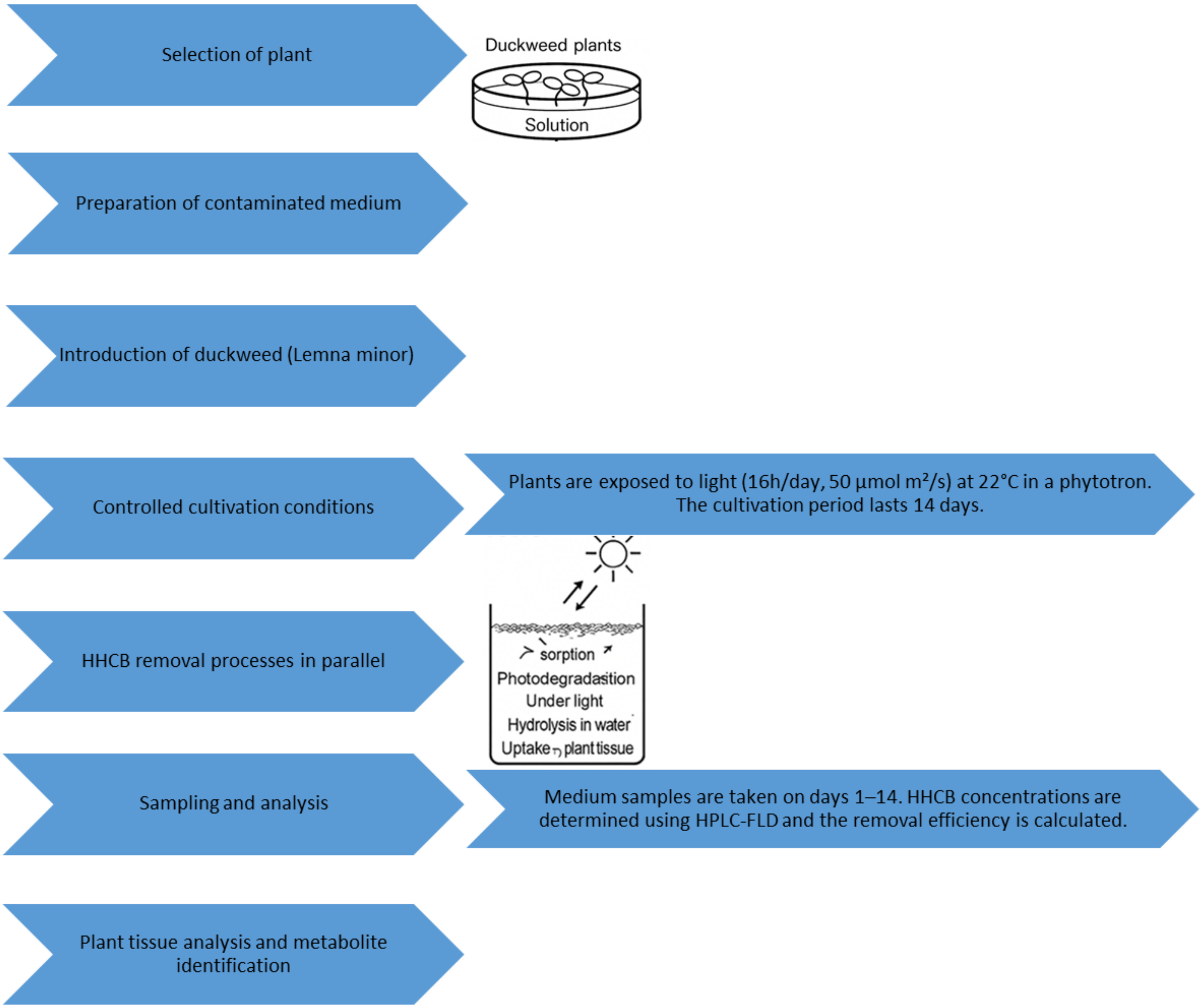

Figure S2. Schematic representation of the laboratory phytoremediation setup and sequential steps in the HHCB removal process using Lemna minor.

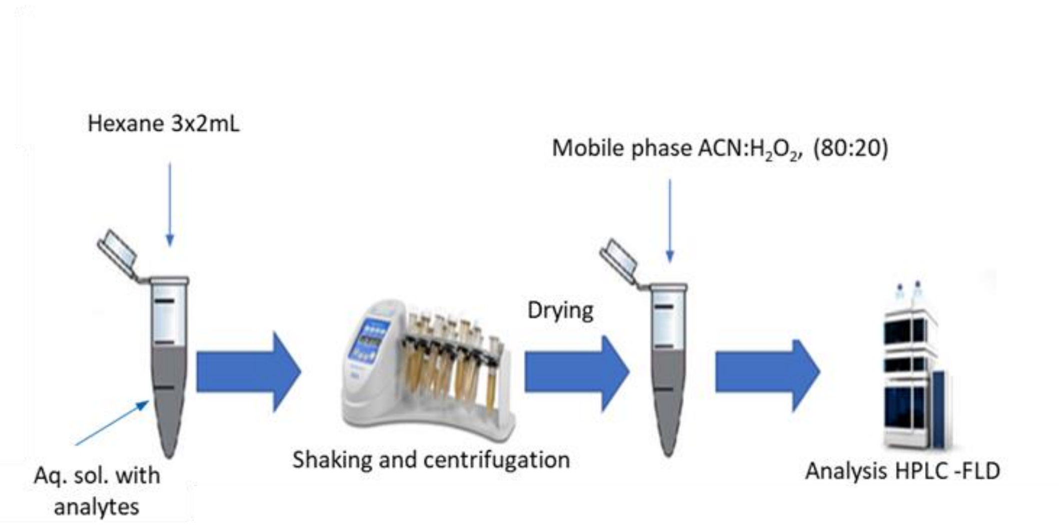

Figure S3. Liquid-liquid extraction (LLE)

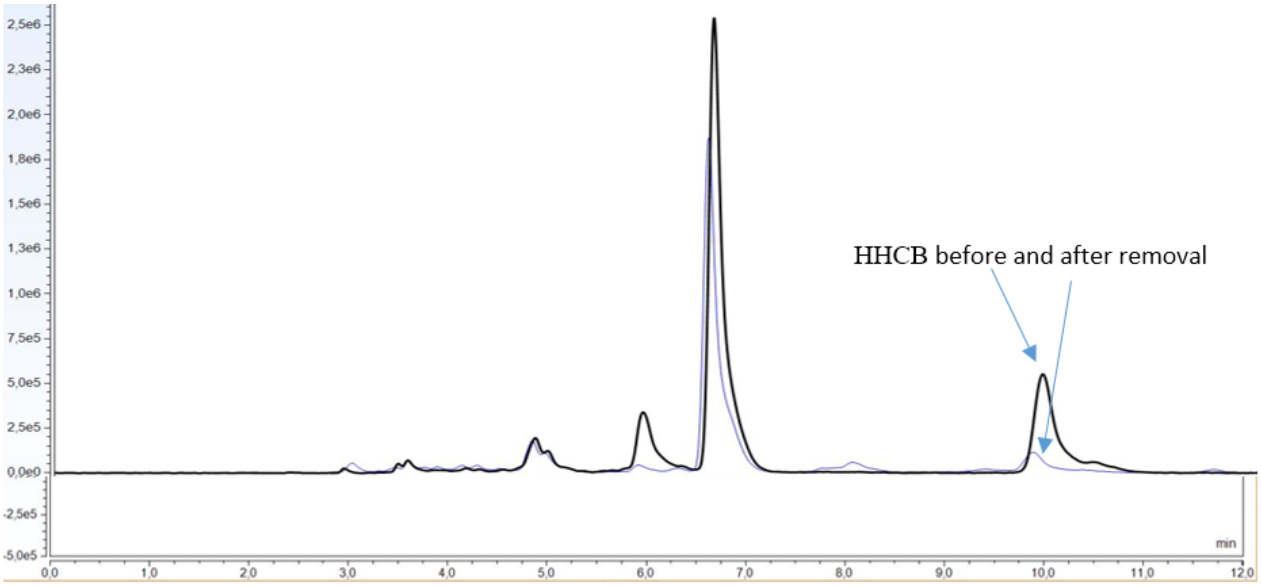

Figure S4. Chromatogram of HHCB(2326 μg·L^{-1}) before and after removal by *Lemna minor* from wastewater.

Table S4 Analytical characteristics of galaxolide determination by HPLC-FLD method

	Laboratory nutrient solution	Synthetic wastewater
Measured Value	Parameter	
Average Standard Curve Equation *y= (a±SD)x + (b±SD)* SD-odchylenie standardowe , n=3	y=(70±5)x +29208 ±3252)	y = (107±4)x + 31902 ± 843
Coefficient of Determination (R^2)	0,9951	0,9630
Repeatability of Slope Coefficient *a* CV [%]	6,4	3,8
Linearity range [$\mu g \cdot L^{-1}$]	2 - 2500	2 - 2500
LOD [$\mu g \cdot L^{-1}$]	0,6	0,7
LOQ [$\mu g \cdot L^{-1}$]	2,0	2,1
Accuracy [%]	94,8 ± 5,9	90,9 ± 5,6

Table S5 Repeatability of the HHCB determination method

Measurement Repeatability (n=6)		
	Laboratory nutrient solution	Synthetic Wastewater
HHCB concentration [$\mu g \cdot L^{-1}$]	517	
Average value of surface area	68648	93770
SD	328	2133
CV [%]	0.5	2.20

S4. Determination of Soluble Proteins

The soluble protein content in Lemna minor tissues was determined following the Bradford method, using bovine serum albumin as the standard [48]. The content of soluble proteins in Lemna minor L. was determined spectrophotometrically (Hitachi U-2800A UV-Vis spectrophotometer; Hitachi High-Tech Science Corporation, Tokyo, Japan). 0.1 g of the plant was weighed into an Eppendorf tube to isolate proteins from plant material. Samples were collected on days 2, 5, 7, and 14 of the experiment. 1 mL of extraction buffer was added, and the samples were homogenised and centrifuged. 200 μL of the supernatant solution was taken into test tubes, and 200 μl of diluted Bradford reagent was added in a 1:4 ratio. The blank contained 200 μl of diluted Bradford reagent and 200 μl of distilled water. The solutions were mixed and then incubated at room temperature. Then, 2.6 mL of distilled water was added to each test tube and mixed. The absorbance measurement was performed at 595 nm 60 min after sample preparation. Protein content was determined based on a calibration curve of bovine albumin serum. For this purpose, 100 mg of bovine albumin was weighed and dissolved in 100 mL of distilled water. Then, solutions with selected concentrations in the 0.3-0.9 $mg \cdot L^{-1}$ range were prepared. The procedure for preparing the solutions was the same as for the tested samples. A curve with the equation $y=0.05x + 0.0065 \pm 0.0011$ was obtained.

S5.Determination of Monosaccharides

The monosaccharide content was estimated spectrophotometrically (Hitachi U-2800A UV-Vis spectrophotometer; Hitachi High-Tech Science Corporation, Tokyo, Japan) according to the Somogyi-Nelson method with modification [49,50]. For this purpose, 0.1 g of Lemna minor L. tissues was homogenised in 1 mL of ethanol and centrifuged. All samples (0.5 mL) were treated with 0.5 mL of copper reagent and placed in a boiling water bath for 20 min. After this time, the samples were cooled with cold water. Then 0.5 mL of arsenomolybdate reagent was added, and after 5 minutes, the extract was diluted in 3.5 mL of water and mixed. The absorbance measurement was performed at 540 nm. The sugar content in the tested samples was calculated using a standard curve. To prepare a standard curve, 100 mg of glucose was weighed and dissolved in 100 mL of ethanol. Then, a series of solutions with selected concentrations in the 0.025-0.3 $mg \cdot L^{-1}$ range were prepared. The determination procedure was the same as for the tested samples.

S6. Determination of Malondialdehyde and H_2O_2 Content

The amount of total malondialdehyde (MDA) as a marker of lipid peroxidation was determined using Heath and Packer's method [44]. 1 mL of 0.1% trichloroacetic acid (TCA) solution was added to 0.1 g of duckweed. Then, the samples were homogenised and centrifuged. 0.5 mL of supernatant was mixed with 2 mL of 0,37% thiobarbiturate acid (TBA) in 15% TCA. The resulting mixture was incubated in bath water for 10 min at 100^{0}C. Then, the extract was cooled and centrifuged for 10 min.

The level of MDA was measured spectrophotometrically at a wavelength of 535 nm, and the nonspecific absorption at 600 nm was subtracted. The content of the MDA-TBA complex was calculated using the molar extinction coefficient at 155 mM cm^{-1}.

The concentration of hydrogen peroxide (H_2O_2) in Lemna minor cells was assessed using spectrophotometry at 390 nm, employing a 1 M potassium iodide (KI) reaction. To determine the levels, a standard curve was established using freshly prepared H_2O_2 solutions, as detailed in [51]. Malondialdehyde (MDA) and H_2O_2 measurements were conducted on day 14 of cultivation.

S7. Quantification of Photosynthetic Pigments

To determine the content of photosynthetic pigments, 0.1 g of Lemna minor was extracted using 10 mL of 90% methanol, and the samples were then incubated at 60°C for 20 minutes. After this period, the samples were filtered, and the absorbance spectra were recorded at wavelengths of 665, 652, and 470 nm against a blank sample of 90% methanol. The concentrations of chlorophyll a (Ca), chlorophyll b (Cb), and carotenoids (Ck) were calculated using the following formulas [52]: Chlorophyll a (mg·L^{-1}) = 16.82 A665 – 9.82 A652; Chlorophyll b (mg·L^{-1}) = 36.92 A652 - 16.54 A665; Carotenoids (mg·L^{-1}) = (1000 A470 – 1.91 Ca – 95.15 Cb)/225.

S8. Determination of the Antioxidant Enzymes Activities

The antioxidant enzymes were extracted using a 50 mM phosphate buffer at pH 7.0, which included 1 mM EDTA, 0.05% Triton X-100, 2% polyvinylpyrrolidone, and 1 mM ascorbic acid. According to the method [53] catalase (CAT) activity was evaluated by monitoring the decrease in absorbance of H_2O_2 at 240 nm. One unit of CAT activity was defined as the amount of enzyme that decomposes 1 μmol of H_2O_2 per mg of soluble protein per minute at 30 °C.

The activity of superoxide dismutase (SOD) was measured by assessing the inhibition of the photochemical reduction of nitroblue tetrazolium (NBT) at 560 nm, as described by Beauchamp and [54]. One unit of SOD activity per mg of protein was defined as the amount of enzyme required to inhibit 50% of the NBT reduction.

APX activity was measured based on the oxidation of ascorbate, quantified as the amount of enzyme that oxidises 1 μmol of ascorbate per mg of soluble protein per minute at 30 °C, following the method by Nakano and Asada (1981) [55].

GR activity was determined by monitoring the oxidation of NADPH at 340 nm (extinction coefficient 6.2 mmol L^{-1} cm^{-1}) at 25 °C, according to the method by Schaedle and Bassham (1977) [56].
